# Diagnostic Accuracy of Red Cell Distribution Width to Platelet Ratio for the Prediction of Liver Fibrosis in Patients With Chronic Liver Disease From Eastern India

**DOI:** 10.7759/cureus.82014

**Published:** 2025-04-10

**Authors:** D Pavan Sai Kumar Rao, Shubhransu Patro, Vibha Sharma, Arushi Choudhary, Shubham Desale, Preetam Nath

**Affiliations:** 1 Department of Medical Gastroenterology, Gleneagles BGS Global Hospitals, Bengaluru, IND; 2 General Medicine, Kalinga Institute of Medical Sciences, Bhubaneswar, IND; 3 Gastroenterology and Hepatology, Kalinga Institute of Medical Sciences, Bhubaneswar, IND

**Keywords:** chronic liver disease, cirrhosis diagnosis, fibroscan, liver cirrhosis, liver fibrosis, liver fibrosis marker, rpr, transient elastography (fibroscan)

## Abstract

Background

Early diagnosis of liver cirrhosis in patients with chronic liver disease (CLD) can help delay/prevent complications and thereby improve survival. The currently available diagnostic modalities for the non-invasive assessment of hepatic fibrosis, especially FibroScan, are costly and not widely available, whereas various non-invasive scores for the assessment of fibrosis are cumbersome. Hence, we aimed to develop an easy and simple score for predicting cirrhosis in patients from Eastern India suffering from CLD with a better diagnostic accuracy.

Methodology

This cross-sectional, observational study was conducted between September 2019 and September 2021 in East India. Our study participants were patients who had CLD of etiologies such as alcohol-related liver disease, non-alcoholic fatty liver disease, chronic viral hepatitis B, chronic viral hepatitis C, primary biliary cholangitis, and autoimmune hepatitis, who had undergone FibroScan of the liver. All demographic details were noted, and the patients were subjected to physical examination, followed by hematological as well as biochemical investigations, including liver function tests. Non-invasive scores (such as aspartate aminotransferase (AST) to platelet ratio index (APRI) and Fibrosis-4 score (FIB-4) and red cell distribution width (RDW) to platelet ratio (RPR)) were computed, and their diagnostic accuracy for prediction of advanced fibrosis and cirrhosis were evaluated by receiver operating characteristic curve (ROC curve) analysis with comparison of area under the ROC curves. Pearson correlation and logistic regression analysis were also performed to study the association of these scores with advanced fibrosis and cirrhosis.

Results

The area under the ROC (AUROC) curve of the APRI score, FIB-4 score, RPR, and RPR × AST for prediction of advanced liver fibrosis was 0.817, 0.799, 0.706, and 0.811, respectively. Similarly, the AUROC of the above scores for the prediction of cirrhosis was 0.889, 0.858, 0.797, and 0.898. However, the product of RPR and AST was superior than APRI and FIB-4 for predicting cirrhosis. An RPR × AST value above the cut-off of 4.818 can help predict liver cirrhosis with 85.7% sensitivity and 85.5% specificity. Pearson correlation and logistic regression analysis also proved the association of these scores with liver fibrosis.

Conclusions

RPR is a simple, inexpensive, and easily available marker for predicting liver cirrhosis. Nevertheless, the variable RPR × AST can predict liver cirrhosis in patients with CLD with even greater diagnostic accuracy.

## Introduction

Cirrhosis of the liver refers to the development of regeneration nodules surrounded by fibrosis and associated with distortion in the liver architecture, as the ultimate result of many chronic liver diseases (CLDs). It can result in several complications, eventually leading to end-stage liver disease and hepatocellular carcinoma. Moreover, liver cirrhosis is one of the leading causes of mortality worldwide. Moreover, it was associated with around 2.4% of all global deaths in 2019 [1]. The fibrosis stage of the liver in CLD is one of the major prognostic factors for the development of liver cirrhosis and other complications, including hepatic decompensating events such as ascites, hepatic encephalopathy, and acute kidney injury [2]. Early diagnosis of fibrosis stage and timely intervention can delay disease progression, avoid or delay the need for liver transplant, and decrease mortality. Liver tissue biopsy is still considered the gold-standard test for the diagnosis of hepatic fibrosis stage; however, it may be associated with complications such as pain in 1%-5%, bleeding, hematoma, and risk of severe complications in 0.57%, which can lead to mortality in 0.009%-0.12% cases [3]. Although liver biopsy examination still has a pivotal role in the diagnosis of CLD, it cannot be utilized widely given its limited availability and risk of potentially fatal complications. The non-invasive modalities for the assessment of liver fibrosis, such as FibroScan/transient elastography (TE), magnetic resonance elastography (MRE), and various serum biomarkers, have high accuracies for the detection of advanced fibrosis as well as liver cirrhosis. However, most of these modalities are expensive and not readily available everywhere except in some tertiary care centers [4-8]. Several serum biomarkers are not liver-specific and may be secreted when other organs are inflamed. Among all, the red blood cell distribution width (RDW) to total platelet count ratio (RPR) has shown a good efficacy in evaluating advanced hepatic fibrosis and cirrhosis in CLD, according to some studies [9]. Hence, we attempted to study the diagnostic accuracy of RPR for the prediction of advanced fibrosis as well as liver cirrhosis.

## Materials and methods

Study design and duration

This hospital-based study with a cross-sectional design was conducted between September 2019 and September 2021 in the Departments of Gastroenterology and General Medicine, Kalinga Institute of Medical Sciences (KIMS), Bhubaneshwar, Eastern India.

Inclusion and exclusion criteria

The study participants were consecutive patients with CLD due to various causes such as alcohol-related liver disease (ALD), non-alcoholic fatty liver disease (NAFLD), chronic viral hepatitis B (CHB), chronic viral hepatitis C (CHC), primary biliary cholangitis (PBC), and autoimmune hepatitis (AIH), who had undergone FibroScan (TE) of the liver during the study period. Fatty liver was defined according to the standard criteria accepted by the American Gastroenterological Association [10], i.e., an increase in hepatic echogenicity with renal echogenicity as a reference. NAFLD was diagnosed when all secondary causes of hepatic steatosis such as consumption of alcohol >20 g/day, having other known liver diseases (hepatitis viruses A to E, autoimmune disease, Wilson’s disease) and those on medications known to induce fatty liver or insulin sensitization such as estrogens, amiodarone, methotrexate, tamoxifen, glitazones, and metformin were excluded. Individuals with a history of any malignancies, congestive cardiac failure, pregnancy, acute hepatitis, chronic liver diseases with ascites (decompensated stage), vascular liver diseases (hepatic venous outflow tract obstruction), hematological disorders such as thalassemia, iron deficiency anemia, and acute inflammatory disorders were also excluded.

Data collection and laboratory investigations

After obtaining written consent from each patient, a detailed history was taken and followed by a thorough physical examination. All participants were subjected to standard laboratory investigations such as complete blood count (CBC), renal function tests (RFTs), liver function tests (LFTs), prothrombin time (PT), international normalized ratio (INR), hepatitis B surface antigen (HBsAg), and anti-hepatitis C virus antibody (snti-HCV). An etiological workup was attempted for all patients. Radiological evaluation was performed using TE (EchoSens FibroScan 502TM) and transabdominal sonography. The patients were categorized into different fibrosis grades (F0 to F4) [11-15] (Table 1). A contrast-enhanced CT scan (with triple phase) of the abdomen was performed whenever required to confirm the diagnosis, along with upper gastrointestinal endoscopy using an Olympus Gastrointestinal Fiberscope-190 gastroscope.

**Table 1:** Fibrosis stage and approximate cutoff values of LSM in FibroScan. LSM: liver stiffness measure; NAFLD: non-alcoholic fatty liver disease; ALD: alcohol-related liver disease

Condition	F0–F1	F2	F3	F4
Chronic hepatitis B and C	2–8	8–10	10–14	>14
Cholestatic liver disease	2–7	7–9	9–17	>17
NAFLD	2–7	7–10	10–14	>14
ALD	2–7	7–11	11–19	>19

The non-invasive scores for fibrosis assessment of the liver, such as Fibrosis-4 score (FIB-4), aspartate aminotransferase (AST) to platelet ratio Index (APRI), RPR, and RPR × AST were computed using the following formulae:

FIB-4 Score = Age (in years) × AST (in U/L)/{platelet (10^9^/L) × ALT1/2 (in U/L)} [16].

APRI Score = {(AST/upper normal limit of AST)/platelet (10^9^/L)} × 100 [16].

RPR = RDW (%)/platelet (10^9^/L) [16].

Statistical analysis

The collected data were entered in Microsoft Excel (2016) (Microsoft Corp., Redmond, WA, USA) and analyzed using SPSS version 22 (IBM Corp., Armonk, NY, USA) [17]. Results were expressed as numbers and frequencies. Relevant statistical tests, such as the chi-square test, unpaired t-test, Pearson’s correlation, regression analysis, and receiver operating curve (ROC), were applied. A p-value of less than 0.05 was considered statistically significant.

Ethical approval and consent

The study protocol was approved by KIMS Institutional Ethical Committee (reference number: KIIT/KIMS/IEC/123/2019). Written informed consent was obtained from all study participants.

## Results

Demographic findings

A total of 193 eligible patients were included in this study. Most patients were in the 41-50-year age group (n = 55, 28.5%), followed by the 51-60-year age group (n = 53, 27.5%). There was a clear male predominance (males: 139, 72%) in the study population. Majority of the patients had comorbidities such as diabetes (76, 39.4%), followed by hypertension (21, 10.9%), dyslipidemia (15, 7.8%), and hypothyroidism (6, 3.1%), whereas bronchial asthma was seen in only 3 (1.6%) participants (Table 2).

**Table 2:** Baseline characteristics of study participants. SD: standard deviation; F0-F1: mild grade of liver fibrosis; F2: moderate liver fibrosis; F3: severe liver fibrosis; F4: cirrhosis of the liver; NAFLD: non-alcoholic fatty liver disease; ALD: alcohol-related liver disease

Variables		N (%) or mean ± SD
Gender	Male	139 (72.1%)
Age	<30 years	24 (12.4%)
	31–40 years	33 (17.1%)
	41–50 years	55 (28.5%)
	51–60 years	53 (27.5%)
	>60 years	28 (14.5%)
Comorbidities	Hypertension	21 (10.9%)
	Diabetes mellitus	76 (39.4%)
	Bronchial asthma	3 (1.6%)
	Hypothyroidism	6 (3.1%)
	Dyslipidemia	15 (7.8%)
Ultrasonography findings	Liver size	13.2 cm ± 2.2 cm
	Spleen size	10.3 cm ± 1.6 cm
Ultrasonography fatty liver grades	0	73 (37.8%)
	1	78 (40.4%)
	2	34 (17.6%)
	3	8 (4.1%)
Coarse liver echogenicity		143 (74.1%)
FibroScan fibrosis grades	F0-F1	109 (56.5%)
	F2	38 (19.8%)
	F3	18 (9.3%)
	F4	28 (14.5%)
Diagnosis (cause of chronic liver disease)	NAFLD	108 (55.9%)
	ALD	36 (18.7%)
	Chronic hepatitis B	44 (22.8%)
	Chronic hepatitis C	3 (1.6%)
	Autoimmune hepatitis	1 (0.5%)
	Primary biliary cholangitis	1 (0.5%)

Laboratory and imaging findings

In the study population, the mean liver size was 13.2 ± 2.2 cm, and the mean splenic size was 10.3 ± 1.6 cm. Among fatty liver grades, the majority had grade 1 fatty liver (78, 40.4%), followed by grade 2 (34, 17.6%), and grade 3 (8, 4.3%). Overall, 73 (37.8%) participants did not have any fatty change in the liver. Coarse echogenicity was observed in 143 (74.1%) participants (Table 2). The patients were categorized into different fibrosis grades (F0 to F4) appropriate for the specific etiologies of CLD as per the liver stiffness measurement (LSM) scores of FibroScan. The majority of the patients had F0-F1 (109, 56.5%) grade of fibrosis, followed by the F2 (38, 19.8%), F3 (18, 9.3%), and F4 (28, 14.5%) grades. Overall, 84 (43.5%), 46 (23.8%), and 28 (14.5%) participants had significant fibrosis of the liver (F2, F3, F4), advanced fibrosis of the liver (F3 and F4), and cirrhosis of the liver (F4), respectively. Among the etiologies of CLD, NAFLD (108, 55.9%) was the most common cause, followed by CHB (44, 22.8%), ALD (36, 18.7%), and chronic hepatitis C (3, 1.6%). One patient had AIH, and PBC was seen in one participant (Table 2).

In the study population, the median and interquartile range (IQR) of AST, alanine aminotransferase (ALT), alkaline phosphatase (ALP), and gamma-glutamyltransferase (GGT) were 35 (IQR = 27-54.5), 36 (IQR = 25-60), 94 (IQR = 73.5-122), and 48 (IQR = 27-78), respectively. The median and IQR of APRI score, FIB-4 score, RPR score, neutrophil-to-lymphocyte ratio (NLR) score, and RPR × AST scores were 0.458 (IQR = 0.302-0.780), 1.359 (IQR = 0.994-2.144), 0.078 (IQR = 0.056-0.1), 2.385 (IQR = 1.688-3.390), and 2.784 (IQR = 1.744-4.774), respectively. In the study population, the median and IQR of the LSM median score and controlled attenuation parameter (CAP) median score were 6.5 (IQR = 5.1-9.8) and 270 (IQR = 219.5-313.5), respectively (Table 3).

**Table 3:** Baseline laboratory and FibroScan parameters of study participants. IQR: interquartile range; AST: aspartate transaminase; ALT: alanine transaminase; ALP: alkaline phosphatase; GGT: gamma-glutamyl transferase; APRI: aspartate transaminase to total platelet count ratio; FIB-4: Fibrosis-4 Index; RPR: red cell distribution width to total platelet count ratio; NLR: neutrophil to lymphocytic ratio; LSM: liver stiffness measurement; CAP: controlled attenuation parameter

Parameters		Median	IQR
AST		35	27–54.5
ALT		36	25–60
ALP		94	73.5–122
GGT		48	27–78
APRI		0.458	0.302–0.780
FIB-4		1.359	0.994–2.144
RPR		0.078	0.056–0.1
NLR		2.385	1.688–3.390
RPR × AST		2.784	1.744–4.774
FibroScan	LSM median (kPa)	6.5	5.1–9.8
	CAP median (dB/m)	270	219.5–313.5

Pearson correlation analysis

Pearson correlations with different fibrosis scores with LSM measured in FibroScan were the highest with FIB-4 (0.57), followed by RPR × AST (0.48), followed by APRI (0.45), liver size (-0.43), RPR (0.36), and spleen size (0.24). NLR did not show any correlation (-0.08) with LSM (Table 4).

**Table 4:** Correlation of fibrosis score with other variables. AST: aspartate transaminase; APRI: aspartate transaminase to total platelet count ratio; FIB-4: Fibrosis-4 Index; RPR: red cell distribution width to total platelet count ratio; NLR: neutrophil to lymphocytic ratio

Pearson correlation with fibrosis	R-value	P-value
FIB-4	0.572	0.04
RPR × AST	0.484	0.17
APRI	0.454	0.23
Liver size	0.433	0.31
RPR	0.36	0.18
Spleen size	0.245	0.36
NLR	0.082	0.28

Logistic regression analysis

The binary logistic regression of cirrhosis showed a significant association (p = 0.03) with FIB-4, with an odds ratio of 1.654, and with liver size, with an odds ratio of 0.56. APRI and RPR × AST did not show any significant association with cirrhosis. Binary logistic regression of advanced fibrosis showed a significant association (p < 0.001) with FIB-4, with an odds ratio of 2.588, and for liver size, with an odds ratio of 0.446. APRI and RPR × AST did not show any significant association with advanced fibrosis. Binary logistic regression of significant fibrosis showed a significant association (p = 0.011) with FIB-4, with an odds ratio of 1.697, and for liver size, with an odds ratio of 0.753. APRI and RPR × AST did not show any significant association with advanced fibrosis (Table 5).

**Table 5:** Binary logistic regression analysis of fibrosis scores for prediction of cirrhosis, advanced fibrosis, and significant fibrosis. AST: aspartate transaminase; APRI: aspartate transaminase to total platelet count ratio; FIB-4: Fibrosis-4 Index; RPR: red cell distribution width to total platelet count ratio; NLR: neutrophil to lymphocytic ratio; OR: odds ratio; 95% CI: 95% confidence interval

Fibrosis grade (dependent or outcome variable)	Fibrosis scores (independent or predictor variable)	P-value	OR	95% CI
Cirrhosis	APRI	0.288	4.12	0.303-56.079
	FIB4	0.033	1.654	1.041-2.626
	RPR × AST	0.329	1.16	0.861-1.561
	Liver size	0	0.566	0.429-0.748
Advanced fibrosis	APRI	0.663	1.777	0.134-23.624
	FIB-4	0.001	2.588	1.478-4.532
	RPR × AST	0.397	1.144	0.838-1.562
	Liver size	0	0.446	0.331-0.602
Significant fibrosis	APRI	0.659	1.594	0.201-12.661
	FIB-4	0.011	1.697	1.127-2.556
	RPR × AST	0.886	1.02	0.781-1.331
	Liver size	0.001	0.753	0.634-0.894

ROC curve analysis

**Figure 1:**
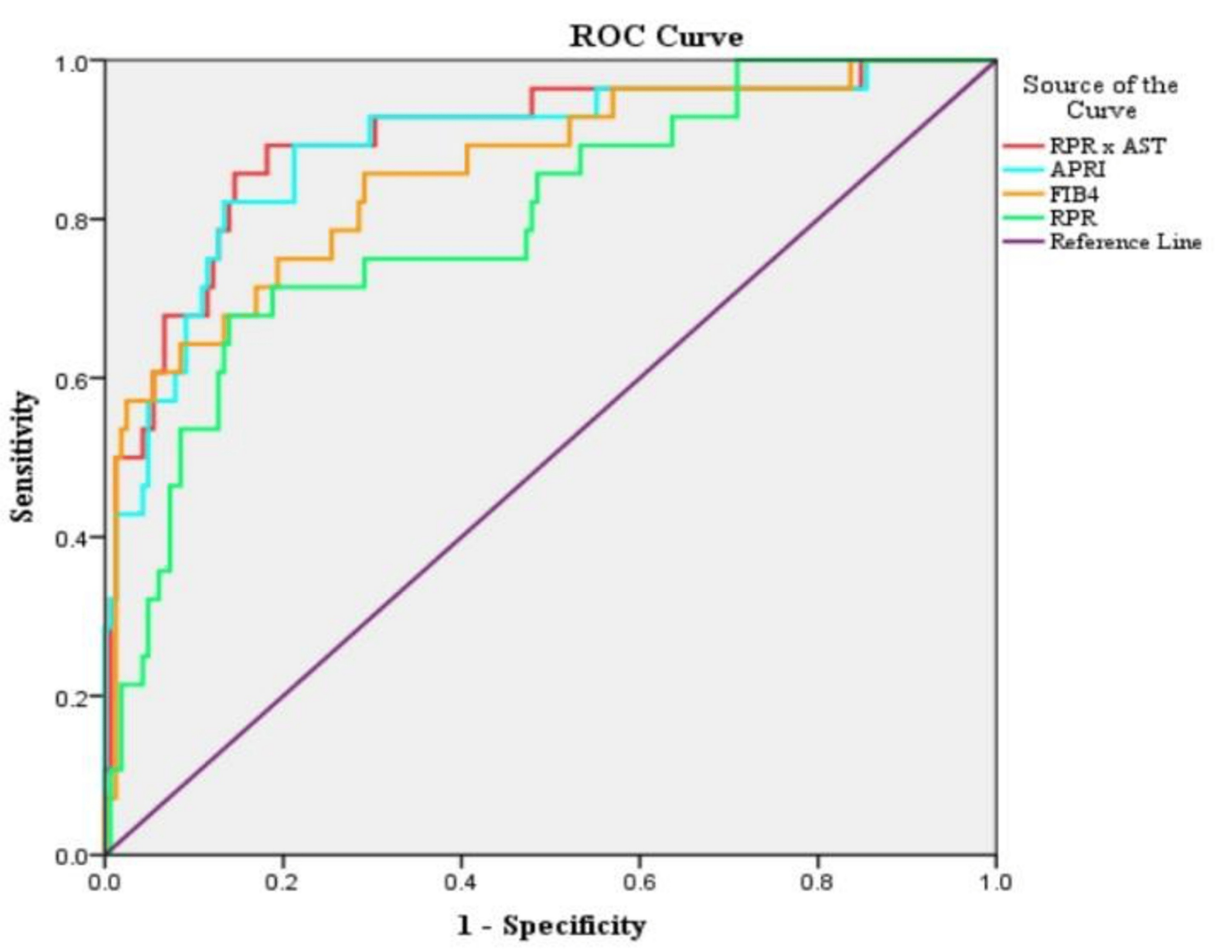
Area under the curve of receiver operating curve (ROC) analysis for various scores predicting cirrhosis. AST: aspartate transaminase; APRI: aspartate transaminase to total platelet count ratio; FIB-4: Fibrosis-4 Index; RPR: red cell distribution width to total platelet count ratio

**Figure 2:**
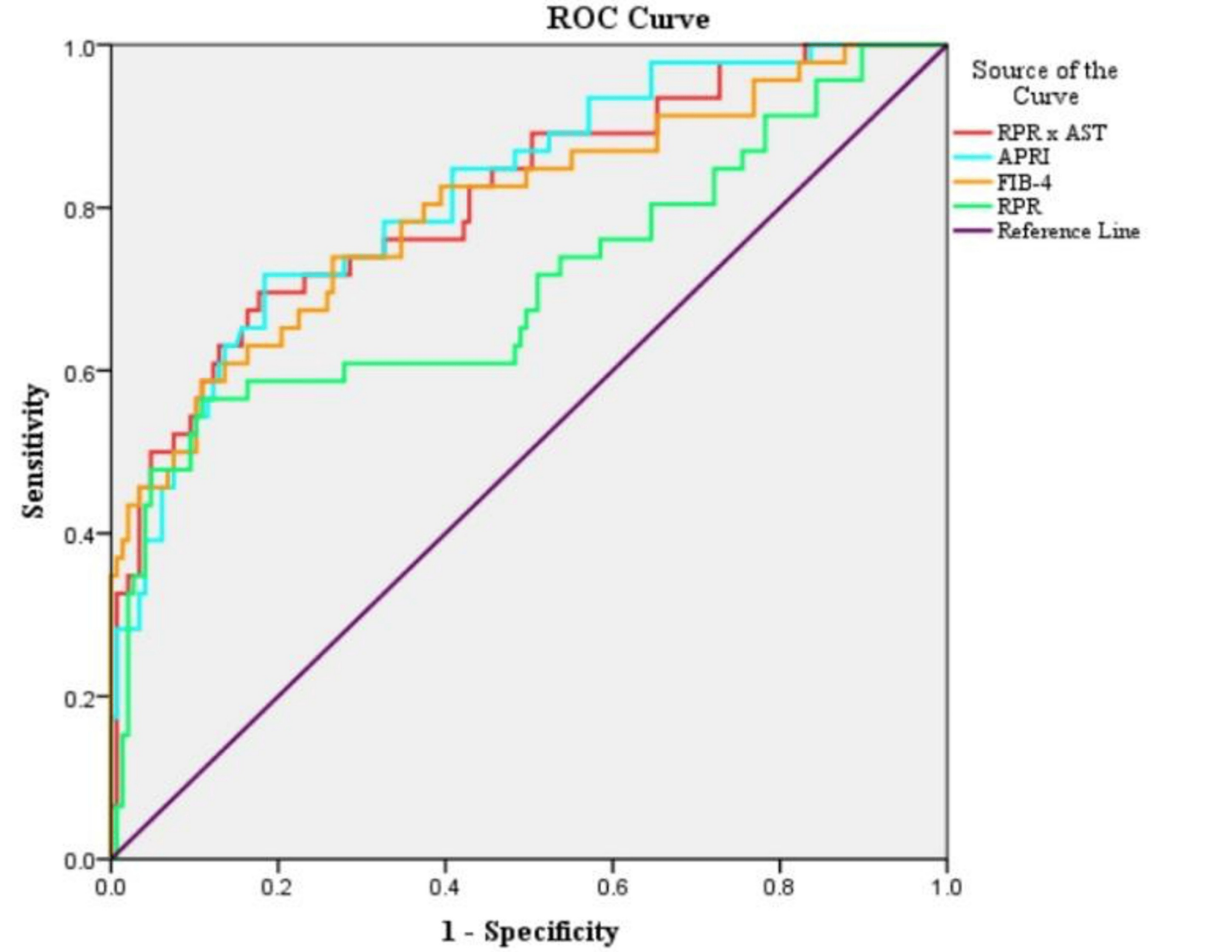
Area under the curve of the receiver operating curve (ROC) analysis for various scores predicting advanced fibrosis. AST: aspartate transaminase; APRI: aspartate transaminase to total platelet count ratio; FIB-4: Fibrosis-4 Index; RPR: red cell distribution width to total platelet count ratio

**Figure 3:**
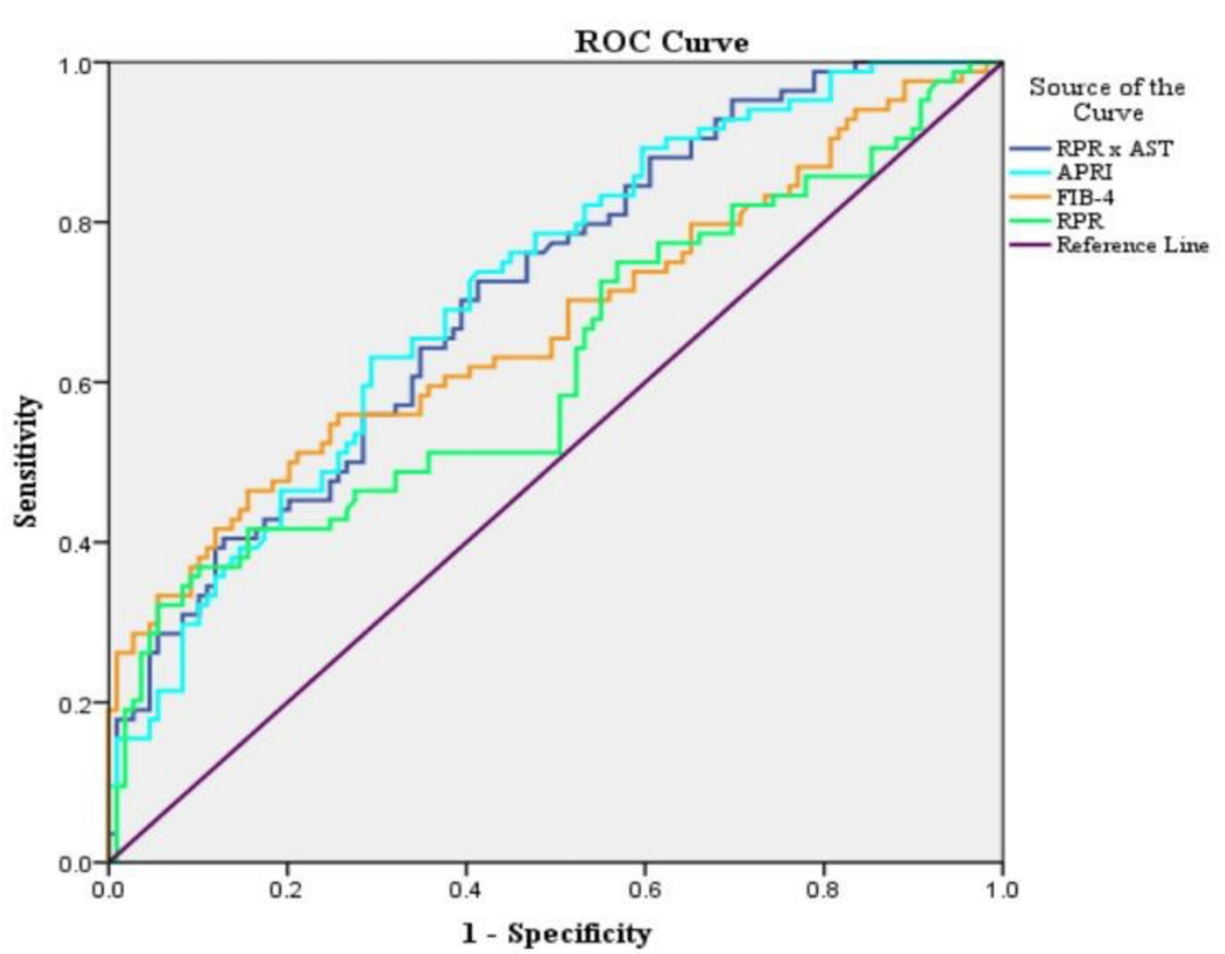
Area under the curves of receiver operating curve (ROC) analysis for various scores predicting significant fibrosis. AST: aspartate transaminase; APRI: aspartate transaminase to total platelet count ratio; FIB-4: Fibrosis-4 Index; RPR: red cell distribution width to total platelet count ratio

In the study population, upon constructing ROC curves for the prediction of liver cirrhosis, RPR × AST showed the highest area under the curve (AUC) at 0.89, followed by APRI at 0.88, FIB-4 at 0.85, and RPR at 0.79. For prediction of advanced liver fibrosis, APRI showed the highest AUC at 0.817, followed by RPR × AST at 0.811, FIB-4 at 0.799, and RPR at 0.706. For significant fibrosis, APRI showed the highest AUC at 0.716, followed by RPR × AST at 0.712, FIB-4 at 0.671, and RPR at 0.622 (Table 6, Figures 1-3).

**Table 6:** Area under the curve (AUC) of receiver operating curve analysis for various scores. AST: aspartate transaminase; APRI: aspartate transaminase to total platelet count ratio; FIB-4: Fibrosis-4 Index; RPR: red cell distribution width to total platelet count ratio; NLR: neutrophil to lymphocytic ratio; OR: odds ratio

Fibrosis grades	Variable	AUC	95% CI	P-value
Cirrhosis	RPR × AST	0.898	0.829-0.968	0.000
	APRI	0.889	0.817-0.962	0.000
	FIB-4	0.858	0.776-0.939	0.000
	RPR	0.797	0.706-0.888	0.000
Advanced fibrosis	APRI	0.817	0.747-0.888	0.000
	RPR × AST	0.811	0.736-0.887	0.000
	FIB-4	0.799	0.719-0.88	0.000
	RPR	0.706	0.608-0.804	0.000
Significant fibrosis	APRI	0.716	0.644-0.787	0.000
	RPR × AST	0.712	0.64-0.783	0.000
	FIB-4	0.671	0.592-0.75	0.000
	RPR	0.622	0.54-0.704	0.004

The cutoff values for APRI, FIB-4 score, RPR, and RPR × AST for the prediction of cirrhosis, advanced, and significant fibrosis have been depicted in Table 8. RPR cutoff values ranged from 0.08 to 0.09, and had sensitivity and specificity ranging from 46% to 67% and 73% to 86%, respectively, for the prediction of cirrhosis, advanced and significant fibrosis. Similarly, an RPR × AST cutoff value above 4.81 could detect liver cirrhosis with a sensitivity of 85.7% and a specificity of 85.4%, respectively (Table 7, Figures 1-3).

**Table 7:** Diagnostic accuracies of various scores for fibrosis grades. AST: aspartate transaminase; APRI: aspartate transaminase to total platelet count ratio; FIB-4: Fibrosis-4 Index; RPR: red cell distribution width to total platelet count ratio; PPV: positive predictive value; NPV: negative predictive value; LR+: positive likelihood ratio; LR-: negative likelihood ratio

Fibrosis grades	Test result variables	Cutoffs	Sensitivity	Specificity	PPV	NPV	LR+	LR-
Cirrhosis	APRI	0.85417	82.14	86.67	51.1	96.6	6.16	0.21
	FIB-4	2.02529	75.00	80.61	39.6	95	3.87	0.31
	RPR	0.10382	67.86	86.06	45.2	94.0	4.87	0.37
	RPR × AST	4.8175	85.71	85.45	50	97.2	5.84	0.17
Advanced fibrosis (F3, F4)	APRI	0.66879	71.74	81.63	55	90	3.91	0.35
	FIB-4	1.72016	73.91	73.47	46.5	90	2.79	0.36
	RPR	0.09923	58.70	83.67	52.9	86.6	3.6	0.49
	RPR × AST	4.3224	69.57	82.31	55.2	89.6	3.93	0.37
Significant fibrosis (F2-F4)	APRI	0.50987	63.10	70.64	62.4	71.3	2.15	0.52
	FIB-4	1.65739	55.95	74.31	62.7	68.6	2.18	0.59
	RPR	0.08963	46.43	72.48	56.5	63.7	1.69	0.73
	RPR × AST	2.7011	70.24	60.55	57.8	72.5	1.78	0.49

## Discussion

In the present study, most patients were in the 41-60-year age group (mean age = 47 years), with a male predominance. Alameri et al. [18] performed a similar study which had a median age of 49 years with a male predominance (53%), which is consistent with the present study. Similarly, Fallatah et al. [19] had 53% males with a mean age of 50.2 years in their study population, which almost coincides with our study population.

In this study, the majority of the participants had diabetes mellitus, followed by hypertension. According to the Indian National Association for Study of the Liver, the majority of NAFLD patients have associated metabolic syndrome with comorbidities such as type 2 diabetes and hypertension [20]. Furthermore, the presence of concomitant comorbidities has been associated with an increased risk of mortality in patients with liver cirrhosis [21]. The majority were diagnosed with NAFLD (108, 52.8%), followed by chronic hepatitis B (44, 22.8%). Ginès et al. [22] observed that around 6%-7% of the adult general population without any known liver disorder had liver fibrosis, and the majority of these individuals had NAFLD, which is consistent with our findings.

In this study, liver size, APRI, FIB-4 Score, and RPR × AST showed significant moderate correlation with LSM scores measured using FibroScan, whereas RPR and spleen size showed a weak correlation. NLR did not show any correlation. The correlation coefficients between fibrosis scores (LSM) on FibroScan and FIB-4 scores (r = 0.5), and APRI (r = 0.51) observed in the study by Fallatah et al. [19] are almost similar to the present study. However, correlation between LSM and FIB-4 scores (r = 0.08), and RPR × AST (r = 0.06) observed by Wang et al. [23] are not consistent with our study findings.

ROC curves showed RPR × AST (highest accuracy), APRI, and FIB-4 to have very good accuracies (AUC = 0.8-0.9) for detecting cirrhosis than RPR, whereas for detection of advanced fibrosis, APRI showed slight predominance over RPR × AST but showed very good accuracy, and FIB-4 and RPR showed good accuracy. Similarly, in detecting significant fibrosis, APRI and RPR × AST showed good accuracy, whereas FIB-4 and RPR showed satisfactory accuracy.

RPR showed good accuracy in detecting advanced fibrosis (0.71), which is similar to the findings of Wu et al. [24], who also reported a similar AUC for RPR. However, many other studies [25-28] reported higher AUC. Table 8 presents different studies with observed cutoff values with respective sensitivities and specificities [11,25-32]. Most studies reported similar findings. The individual components of RPR (RDW and platelet count) have already been shown to be associated with liver fibrosis in previous studies. Although the association between RDW and the degree of liver fibrosis is well known, the exact mechanism for linking these two variables is poorly understood. Various abnormalities, such as chronic inflammation leading to erythrocyte fragmentation, oxidative stress, poor nutritional condition, and abnormality of erythropoietin function, can cause elevation in RDW in patients with CLD [33]. Furthermore, thrombocytopenia is known as a common hematological complication in CLD [34].

**Table 8:** Comparison of RPR cut-offs in different studies. ALD: alcohol-related liver disease; NAFLD: non-alcoholic fatty liver disease; AIH: autoimmune hepatitis; PBC: primary biliary cholangitis; RPR: red cell distribution width to total platelet count ratio

Fibrosis grade	Study	RPR cut-off	Sensitivity	Specificity	Study population
Cirrhosis	Present study	0.10382	67.86	86.06	All (NAFLD, ALD, chronic viral hepatitis, AIH, and PBC)
	Cengiz et al. [25]	0.11	83.3	93.2	NAFLD
	Li et al. [11]	0.16	73.7	93	Chronic hepatitis B
	Chen et al. [29]	0.07	75	76.3	Chronic hepatitis B
	Huang et al. [27]	0.09	60.2	78.6	Chronic hepatitis B
	Lee et al. [26]	0.06	66	67.6	Chronic hepatitis B
	Taefi et al. [30]	0.08	82.7	61	Chronic hepatitis B
Advanced fibrosis	Present study	0.09923	58.7	83.67	All (NAFLD, ALD, chronic viral hepatitis, AIH, and PBC)
	Cengiz et al. [25]	0.07	76.5	82.1	NAFLD
	Huang et al. [27]	0.07	75.2	64.5	Chronic hepatitis B
	Jiang et al. [31]	0.14	49.1	95.8	PBC
	Koksal et al. [28]	0.07	93	67	All (NAFLD, ALD, chronic viral hepatitis, AIH, PBC)
	Lee et al. [26]	0.06	81.3	71.3	Chronic hepatitis B
	Wu et al. [29]	0.09	73	67.5	Chronic hepatitis B
Significant fibrosis	Present study	0.08963	46.43	72.48	All (NAFLD, ALD, chronic viral hepatitis, AIH, PBC)
	Cengiz et al. [25]	0.07	47.7	84.8	NAFLD
	Li et al [11]	0.08	39.2	90	Chronic hepatitis B
	Huang et al. [27]	0.07	69.6	68	Chronic hepatitis B
	Karagoz et al. [32]	0.07	44.8	87	Chronic hepatitis B
	Koksal et al. [28]	0.07	56	61	All (NAFLD, ALD, chronic viral hepatitis, AIH, PBC)

Study strengths

In this study, we compared the diagnostic accuracy of RPR among different etiologies of CLD rather than a single group of etiology. Furthermore, we excluded confounding factors that can influence RDW, such as anemia. We considered a new parameter, RPR × AST, which showed excellent accuracy in the prediction of liver fibrosis compared to other preexisting scores. In our study, we compared the parameters using FibroScan, which has good accuracy in the prediction of liver fibrosis in CLD but is only available in tertiary care centers.

Study limitations

This was a single-centered, hospital-based study with a cross-sectional design. In addition, a smaller sample size may limit the generalizability of the findings. We did not confirm liver fibrosis with liver biopsy, which is considered to be the gold-standard test for the diagnosis of hepatic fibrosis. In our study population, most were not followed further after diagnosis, and most participants were in the F0-F1 category. Lastly, although RPR was comparable with other scores for predicting advanced fibrosis as well as cirrhosis in patients with CLD, it was slightly inferior to other scores. However, it is easily available and a simple score to predict fibrosis in CLD. Moreover, the new score RPR × AST has been observed to be better.

## Conclusions

Our study demonstrates that RPR is a cost-effective, easily accessible, and non-invasive marker for predicting liver cirrhosis and advanced fibrosis in patients with CLD. However, RPR × AST exhibited superior diagnostic accuracy compared to RPR alone, APRI, and FIB-4, making it a valuable tool for clinicians assessing liver fibrosis in resource-limited settings. Given that invasive methods such as liver biopsy pose risks and non-invasive imaging techniques such as FibroScan are expensive and not widely available, serum-based fibrosis indices such as RPR and RPR × AST can be an important alternative for initial screening and follow-up. These may be incorporated into clinical practice to improve early detection, thereby allowing for timely interventions and improved patient outcomes. In conclusion, while RPR alone is a useful marker, RPR × AST provides superior diagnostic accuracy and may be a reliable non-invasive alternative for detecting cirrhosis and advanced fibrosis. With further validation, these markers could contribute significantly to risk stratification, treatment planning, and disease monitoring in CLD patients.
